# Enterovirus-Human Rhinovirus: A Rare Cause of Acute Respiratory Distress Syndrome

**DOI:** 10.1177/2324709617728526

**Published:** 2017-09-05

**Authors:** Parita Soni, Anand Rai, Nidhi Aggarwal, Stephan Kamholz, Taek Yoon, Yizhak Kupfer

**Affiliations:** 1 Maimonides Medical Center, Brooklyn, NY, USA

**Keywords:** acute respiratory distress syndrome, ARDS, enterovirus/human rhinovirus, critical care medicine, infectious diseases, medical education, pulmonary, pneumonia

## Abstract

A 22-year-old Asian woman presented with respiratory distress, cough, and wheezing for 1 week. Prior history included asthma and Turner syndrome. On presentation to the emergency department, the patient was hypotensive, tachycardic, tachypneic, with an oxyhemoglobin saturation in the mid 80% range while breathing ambient air. Chest radiograph revealed pulmonary vascular congestion and a left lower lobe infiltrate. Endotracheal intubation, mechanical ventilation, and vasopressors were initiated. Empiric therapy for community-acquired pneumonia was administered utilizing broad-spectrum intravenous antibiotics. Routine sputum culture was negative for pathogens. Nasopharyngeal swab submitted for multiplex amplified nucleic acid testing yielded enterovirus-human rhinovirus (EV-HRV). Thus, the diagnosis of EV-HRV pneumonia complicated by acute respiratory distress syndrome (ARDS) was established. Multiple attempts to wean from the ventilator were unsuccessful, and a tracheostomy was performed. This report highlights EV-HRV as a cause of severe ARDS and prolonged respiratory failure in adults.

## Introduction

Enteroviruses are single-stranded RNA viruses of the picornavirus family that mainly affect the pediatric population. It is one of the rare cause of acute respiratory distress syndrome (ARDS) in adults. When enteroviruses infects adults, the severity and the disease course varies widely. We report a young woman who developed ARDS secondary to enterovirus-human rhinovirus (EV-HRV) infection.

## Case Presentation

A 22-year-old Asian woman presented to the emergency department (ED) complaining of respiratory distress, cough, and wheezing of 1 week duration. Her past medical history included asthma, Turner syndrome, scoliosis treated with spine surgery, and Harrington rod implantation. The patient was also diagnosed with cerebral palsy in her early childhood. Due to this, she had swallowing difficulties leading to failure to thrive. Hence, her nutritional requirements were managed via percutaneous endoscopic gastrostomy. The patient’s mother had noticed yellowish green thick respiratory secretions for several days. Her last hospitalization was 6 months prior because of bronchitis.

**Figure 1.**
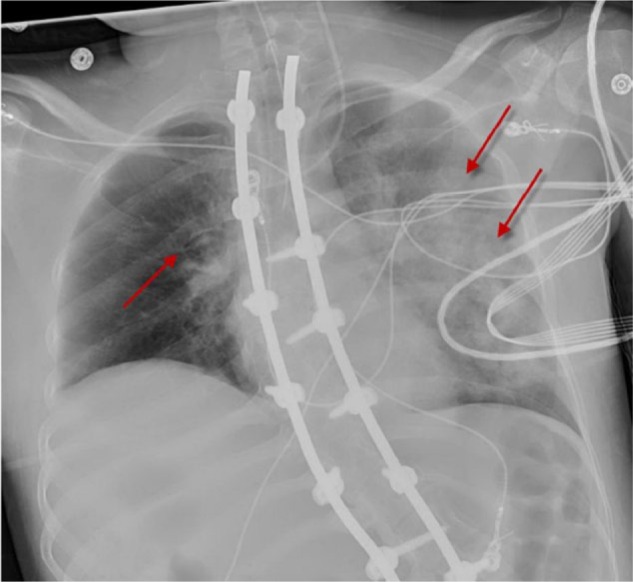
Chest radiograph demonstrating diffuse bilateral infiltrates (left > right) and marked pulmonary vascular congestion (arrows).

In the ED, her blood pressure was 73/37 mm Hg, respiratory rate 32/min, and SpO_2_ of 80% breathing ambient air. Arterial blood gas values indicated hypoxic respiratory failure with PaO_2_ 32 mm Hg and FiO_2_ 0.21 (PaO_2_/FiO_2_ ratio = 152). Complete blood count revealed white blood cells 15 700/µL with left shift. Duplex ultrasound scan of the bilateral lower extremities was negative for thrombosis. Chest radiograph revealed pulmonary vascular congestion and a left lower lobe infiltrate (Figure 1). Transthoracic echocardiogram (ECHO) performed previously was normal. Endotracheal intubation and mechanical ventilation were initiated, and vasopressors were titrated to maintain mean arterial pressure ≥60 mm Hg.

Empiric treatment for presumed community-acquired pneumonia included azithromycin and ceftriaxone. Bedside fiberoptic bronchoscopy revealed no airway abnormalities. Sputum microbiologic examination was negative for bacterial pathogens. The bronchoalveolar lavage fluid was negative for any viral, bacterial, or fungal organisms. Multiplex amplified nucleic acid testing of a nasopharyngeal swab specimen was positive for EV-HRV. The diagnosis of EV-HRV pneumonia causing ARDS was established.

## Outcome

A tracheostomy was performed on day 13 after multiple unsuccessful attempts to wean from mechanical ventilation. The patients was transferred to a rehabilitation facility.

## Discussion

EV-HRVs are positive-sense, single-stranded-RNA viruses of approximately 7200 bp that are members of the family picornaviridae and the genus *enterovirus*.^1^ Three genetically distinct HRV groups (A, B, and C) have been isolated based on advanced molecular techniques.^1^ EV-HRVs commonly causes mild upper respiratory tract illnesses. However, in some instances they can cause severe and potentially fatal conditions, such as aseptic meningitis, encephalitis, acute asthma exacerbations, bronchiolitis, viral pneumonia, myocarditis, acute flaccid paralysis, and hand-foot-and-mouth disease.^2-5^ The severity of respiratory symptoms in EV-HRV infection depends on the production of numerous pro-inflammatory cytokines and chemokines such as interleukin (IL)-1, IL-6, and IL-8.^1^ These pro-inflammatory molecules causes airway inflammation.

EV-HRV infection can cause ARDS. ARDS is characterized by diffuse inflammation of the lungs leading to severe respiratory distress and hypoxemia refractory to oxygen therapy. ARDS is a clinical phenotype that can be triggered by various etiologies such as infection, trauma, and sepsis. It is associated with mortality rates ranging from 26% to 58%.^6-8^ EV-HRV infection leading to ARDS is common in the pediatric population, especially in children with a history of asthma or wheezing.^9^ The Centers for Disease Control and Prevention reported 1121 laboratory-confirmed cases of enteroviruses in the United States from mid-August to December 2014.^9^ Of these 1121 cases with enteroviruses infections, almost all of them occurred in children, mainly those with a history of asthma or wheezing.^9^

Very few reports of EV-HRV causing severe ARDS in adults exist. The severity and the disease course in EV-HRV-infected adults varies widely; however, the usual manifestation is a mild respiratory illness. The pathogenesis of the severe respiratory illness caused by EV-HRV is not clear.^5,10^ Our patient demonstrates that EV-HRV can cause severe ARDS and prolonged respiratory failure in a young adult. Farrell et al reported a patient who required mechanical ventilation for 32 days.^11^ Early identification of EV-HRV infection using multiplex polymerase chain reaction techniques applied to pharyngeal swab specimens may obviate invasive diagnostic procedures and limit the use of antimicrobial agents in such patients. We suggest that enterovirus infection should be considered in the differential diagnosis of pneumonia causing severe ARDS in patients regardless of age.
